# Mother’s education and offspring asthma risk in 10 European cohort studies

**DOI:** 10.1007/s10654-017-0309-0

**Published:** 2017-09-19

**Authors:** Kate Marie Lewis, Milagros Ruiz, Peter Goldblatt, Joana Morrison, Daniela Porta, Francesco Forastiere, Daniel Hryhorczuk, Oleksandr Zvinchuk, Marie-Josephe Saurel-Cubizolles, Sandrine Lioret, Isabella Annesi-Maesano, Martine Vrijheid, Maties Torrent, Carmen Iniguez, Isabel Larranaga, Margreet W. Harskamp-van Ginkel, Tanja G. M. Vrijkotte, Jana Klanova, Jan Svancara, Henrique Barross, Sofia Correia, Marjo-Riitta Jarvelin, Anja Taanila, Johnny Ludvigsson, Tomas Faresjo, Michael Marmot, Hynek Pikhart

**Affiliations:** 1 Research Department of Epidemiology and Public Health, University College London, 1-19 Torrington Place, London, WC1E 6BT UK; 2 Research Department of Epidemiology and Public Health, UCL Institute of Health Equity, University College London, London, UK; 3 Department of Epidemiology, Lazio Regional Health System, Rome, Italy; 4 Center for Global Health, University of Illinois College of Medicine, Chicago, IL USA; 5 Institute of Pediatrics, Obstetrics, and Gynecology, National Academy of Medical Sciences of Ukraine, Kiev, Ukraine; 6 Institut National de la Santé et de la Recherche Médicale (INSERM), UMR 1153, Obstetrical, Perinatal and Pediatric Epidemiology Research Team (Epopé), Center for Epidemiology and Statistics, Sorbonne Paris Cité, DHU Risks in Pregnancy, Paris Descartes University, Paris, France; 7 Institut National de la Santé et de la Recherche Médicale (INSERM), UMR 1153, Early Origin of the Child’s Health and Development Team (ORCHAD), Center for Epidemiology and Statistics, Sorbonne Paris Cité, Paris Descartes University, Paris, France; 8 Pierre Louis Institute of Epidemiology and Public Health (iPLESP), UMR 1136, Epidemiology of Allergic and Respiratory Diseases (EPAR), Paris, France; 9 ISGlobal, Center for Research in Environmental Epidemiology (CREAL), Barcelona, Spain; 10 Universitat Pompeu Fabra (UPF), Barcelona, Spain; 11 Spanish Consortium for Research on Epidemiology and Public Health (CIBERESP), Barcelona, Spain; 12 IB-Salut Menorca Health Area, Balearic Islands, Spain; 13 Joint Research Unit of Epidemiology and Environmental Health, FISABIO – Universitat Jaume I – Universitat de Valéncia, Castellón de la Plana, Spain; 14 Public Health Department of Gipuzkoa, Gipuzkoa, Spain; 15 BIODONOSTIA Health Research Institute, San Sebastian, Spain; 16 Department of Public Health, Amsterdam Public Health Research Institute, Academic Medical Center, University of Amsterdam, Amsterdam, The Netherlands; 17 Research Centre of Toxic Compounds in the Environment (RECETOX), Faculty of Science, Masaryk University, Brno, Czech Republic; 18 Institute of Biostatistics and Analyses (IBA), Masaryk University, Brno, Czech Republic; 19 EPIUnit – Institute of Public Health, University of Porto, Porto, Portugal; 20 Department of Clinical Epidemiology, Predictive Medicine and Public Health, University of Porto Medical School, Porto, Portugal; 21 Department of Epidemiology and Biostatistics, MRC Health Protection Agency (HPE), Centre for Environment and Health, School of Public Health, Imperial College London, London, UK; 22 Biocenter Oulu, University of Oulu, Oulu, Finland; 23 Unit of Primary Care, Oulu University Hospital, Oulu, Finland; 24 Faculty of Medicine, Center for Life Course Health Research, University of Oulu, Oulu, Finland; 25 Division of Pediatrics, Department of Clinical and Experimental Medicin, Linköping University, Linköping, Sweden; 26 Department of Medicine and Health, Community Medicine/General Practice Faculty of Health Sciences, Linköping University, Linköping, Sweden

**Keywords:** Asthma, Children, Cohort studies, Maternal education, Socioeconomic position, Disease risk

## Abstract

Highly prevalent and typically beginning in childhood, asthma is a burdensome disease, yet the risk factors for this condition are not clarified. To enhance understanding, this study assessed the cohort-specific and pooled risk of maternal education on asthma in children aged 3–8 across 10 European countries. Data on 47,099 children were obtained from prospective birth cohort studies across 10 European countries. We calculated cohort-specific prevalence difference in asthma outcomes using the relative index of inequality (RII) and slope index of inequality (SII). Results from all countries were pooled using random-effects meta-analysis procedures to obtain mean RII and SII scores at the European level. Final models were adjusted for child sex, smoking during pregnancy, parity, mother’s age and ethnicity. The higher the score the greater the magnitude of relative (RII, reference 1) and absolute (SII, reference 0) inequity. The pooled RII estimate for asthma risk across all cohorts was 1.46 (95% CI 1.26, 1.71) and the pooled SII estimate was 1.90 (95% CI 0.26, 3.54). Of the countries examined, France, the United Kingdom and the Netherlands had the highest prevalence’s of childhood asthma and the largest inequity in asthma risk. Smaller inverse associations were noted for all other countries except Italy, which presented contradictory scores, but with small effect sizes. Tests for heterogeneity yielded significant results for SII scores. Overall, offspring of mothers with a low level of education had an increased relative and absolute risk of asthma compared to offspring of high-educated mothers.

## Introduction

Asthma is a chronic disease of the bronchial tubes in the lungs, which typically develops in childhood, and is characterised by wheezing, breathlessness, chest tightness and coughing [1–3]. Children with this condition face increased absence from school, reduced family life participation and lower quality of life compared to non-asthmatic peers [4]. Asthma is one of the most common diseases amongst children worldwide [1] and is particularly prevalent in Western Europe (9.7% asthma ever in 6–7 year olds) [5]. With the appropriate measures in place most cases of asthma can be controlled, but no proven prevention or cure currently exist [1, 6]. With financial costs for asthma in EU countries estimated at €72.2 billion per annum, the case for improved understanding is strong [7].

The causes of childhood asthma are multifaceted, with genetic and environmental exposures increasing susceptibility to the development of this condition [5, 8]. Known early life risk factors include preterm birth, infant weight gain and adiposity, in utero and post-natal tobacco smoke exposure, household damp or mould, lower respiratory infections and pollutants such as NOx, sulphur dioxide and particulate matter [8–12]. As socioeconomically deprived groups face increased exposure to these health damaging risk factors, grasping how social determinants impact on asthma represents a broader framework for establishing preventative measures. Prospective cohort studies in Sweden, the UK and the Netherlands have previously linked parental income, occupation and education to the increased development and severity of childhood asthma [13–17]; however, this evidence is restricted to a handful of European countries and has limited comparability due, in part, to the use of inconsistent socioeconomic position indictors [18].

This research contributes to current understanding through analysis of maternal education, arguably the most important social determinant of early child health [19], as a risk factor for early childhood asthma across Europe. Europe is highly diverse, with wide variation in social, economic and health policies and outcomes between and within countries [20, 21]. Comparing differences in asthma inequities using a common indicator across the continent will help to decipher contextual factors and enable further consideration of the pathways to asthma development. Early childhood is a crucial developmental period of life and given that asthma trajectories can be set in place as early as 6/7 years old, an optimal time for intervention [17, 18]. We hypothesised that social inequities in asthma risk would differ between European countries, but broadly follow an inverse relationship with mother’s education level.

## Methods

### Data sources

Drawing on research by the DRIVERS for Health Equity research programme [22], the current study used data from 10 European birth cohort studies (Box 1). In total, complete data was available for 47 099 singleton children born between June 1985 and January 2008. The sample comprised of cohorts (in descending order of size) from: England and Wales (UK-MCS, *n* = 13 829); Southeast Sweden (SE-ABIS, *n* = 8308); Oulu and Lapland, Finland (FI-NFBC, *n* = 6892); Porto, Portugal (PT-G21, *n* = 6486); Amsterdam, the Netherlands (NL-ABCD, *n* = 3387); Brno and Znojmo, Czech Republic (CZ-CELSPAC, *n* = 2801); Gipuzkoa, Menorca, Sabadell and Valencia, Spain (ES-INMA, *n* = 1843); Kyiv, Dneprodzerzhinsk (now Kamianske) and Mariupol, Ukraine (UA-FCOU, *n* = 1757); Nancy and Poitiers, France (FR-EDEN, *n* = 1159); and Rome, Italy (IT-GASPII, *n* = 637).

**Box 1:** Cohort study locations, names and cohort members’ years of birth

CZ-CELSPAC:	Czech Republic-the European longitudinal study of pregnancy and childhood, 1991–1992
FI-NFBC:	Finland-Northern Finland birth cohort 1985/1986 study
FR-EDEN:	France-the mother–child study of pre- and postnatal determinants of child growth, development, and health, 2003–2006
IT-GASPII:	Italy-the gene and environment prospective study on infancy in Italy, 2003–2004
NL-ABCD:	The Netherlands-Amsterdam born children and their development study, 2003–2004
PT-G21:	Portugal-the generation XXI study, 2005–2006
ES-INMA:	Spain-the environment and childhood project, 1997–2008
SE-ABIS:	Sweden-all babies in Southeast Sweden, 1997–1999
UA-FCOU:	Ukraine-the family and children of Ukraine study, 1992–1996
UK-MCS:	United Kingdom-Millennium cohort study, 2000–2001

Ethical approval and participant consent had been approved by each cohort study before data collection, and no further ethical approval was required for the current study. Anonymisation and extraction of the relevant data was performed by researchers from each cohort study before combining with other datasets for analyses. For further cohort details, see previous research [22].

### Study variables

The definition of childhood asthma varied across the 10 datasets. SE-ABIS retrieved doctor diagnoses directly from primary care and hospital records at age 5 (ICD-10 code J45). All other studies determined asthma presence (yes/no) by mothers’ answer to a survey question, which asked whether their child had: had a doctor diagnosis of asthma (FR-EDEN age 5, UA-FCOU age 7, PT-G21 age 4/5); asthma as ‘a long term illness’ (FI-NFBC age 7/8) or an ‘allergic disease’ (CZ-CELSPAC age 3); ever had asthma (NL-ABCD age 7/8, IT-GASPII age 7, UK-MCS age 5); or had asthma in the last 12-months (ES-INMA age 3/4). Each cohort study ascertained all other information relevant to the current analyses through mother’s self-report prior to their child’s birth, shortly after birth or when the child was 9-months old (UK-MCS only).

Maternal education level was determined with a variety of questions, including years (FI-NFBC; FR-EDEN; PT-G21; NL-ABCD) and stage (ES-INMA; IT-GASPII; SE-ABIS; UA-FCOU) of schooling completed and highest academic qualification (UK-MCS). CZ-CELSPAC and UA-FCOU enquired about highest education level out of the parents and grandparents. Education levels across countries were converted to the same scale using the international Standard Classification of Education (ISCED 97) [23]. We banded the seven levels of the ISCED 97 into three: low, up to lower secondary education (ISCED 0-2); medium, upper secondary education (ISCED 3); and high, post-secondary education and higher (ISCED 4-6).

Several potential confounding factors were present across all cohorts and were therefore included as covariates in the final models. Maternal age at child’s birth (grouped into <30/≥30 years old). Sex of the child (male/female). Parity, which we dichotomised into yes (nulliparous) or no (multiparous) by mother’s report of whether the current birth was their first live-born child. Maternal ethnicity, dichotomised into prevalent ethnicity of the country/region–defined as Caucasian White (FR-EDEN; IT-GASPII), White (UK-MCS), European (CZ-CELSPAC) or by country of birth (NL-ABCD; ES-INMA)–or minority ethnicity for all women not meeting the definition. Smoking during pregnancy (yes/no) based on the following definitions: ≥one cigarette a day during pregnancy (ES-INMA); smoking throughout pregnancy (UK-MCS); smoking regularly in last 6 (CZ-CELSPAC) or 12 months (UA-FCOU); smoked in 1st or 3rd trimester (PT-G21); smoked at the beginning of pregnancy (FI-NFBC); or smoked at all during pregnancy (IT-GASPII; FR-EDEN; NL-ABCD; SE-ABIS). Non-singleton children (n = 1399) were excluded from further analyses.

### Data analysis

All analyses were conducted in Stata 14 [24]. Missing variables data ranged from 3.2% (sex) to 27.7% (asthma) and missingness in the outcome variable was significant related to lower education level and smoking in pregnancy (*p* < 0.001). To enable comparison across cohorts, cases without complete data for all study variables were excluded from analyses. Sampling and attrition survey weights, available for UK-MCS, and generic weights for non-MCS cohorts (probability weight = 1*)* were applied to all analyses [18]. The distribution of mothers’ education for each cohort was age-standardised using the WHO European Standard Population [25].

Prevalence of asthma by mother’s education was calculated for each cohort. The *χ* ^*2*^ test for trend assessed linearity across educational groups with the exception of UK-MCS, to which linear regression was applied to survey weighted data (and compared to unweighted *χ* ^*2*^ test results). The Relative and Slope Indices of Inequality (RII/SII) were used to compute cohort-specific and total differences in asthma outcome [22, 26, 27]. RII represents the relative change in asthma outcome between highest and lowest education group (the prevalence ratio), whilst the SII is the absolute change (the prevalence difference). A score higher than 1 (RII) and 0 (SII) indicates inequity, and the higher the score the greater the magnitude of the inequity.

RII and SII scores, adjusted for child sex, first birth, mother’s age at birth, mother’s ethnicity and maternal smoking status during pregnancy were determined for each cohort. We pooled the results from each cohort using meta-analysis procedures to obtain mean RII and SII scores at the European level. Assuming between-cohort heterogeneity, we applied a random effects model. The *I* ^*2*^ measure was used to test the extent to which heterogeneity was present. Lastly, we conducted sensitivity analysis comparing the results of fixed and random effects upon the models.

## Results

Table 1 presents characteristics of the final sample. Of the 47 099 children, 47.9% were female, 50.9% of mothers were younger than 30 at their child’s birth and for 46.3% this was their first live birth. UK-MCS and ES-INMA had the highest rates of smoking in pregnancy, 33.0 and 29.7% respectively, compared to 7.8% in NL-ABCD and 8.8% in SE-ABIS. The proportion of mothers from ethnic minorities ranged from 0% (FI-NFBC; SE-ABIS; UA-FCOU) to 14.5% (NL-ABCD). The gap between proportion of mothers in low and high ISCED education levels was largest in IT-GASPII (13.2 vs. 36.0%) and PT-G21 (47.2 vs. 25.4%). Asthma was identified in 3737 (7.9%) children with the prevalence ranging from 1.7% (UA-FCOU) to 14.4% (UK-MCS) across cohorts. As shown in Table 2, the unadjusted trend of increased asthma prevalence with lower maternal education level was strongest in the UK-MCS (*p* < 0.001), NL-ABCD (*p* < 0.001) and FR-EDEN (*p* = 0.008) cohorts. A weaker trend was noted for SE-ABIS (*p* = 0.031). IT-GASPII showed a reverse pattern, with asthma more prevalent in the highest education level; however, this results showed no significant trend (*p* = 0.60). There was little to no evidence of an educational trend across all other cohorts, which, in some cases, may be due to a lack of statistical power to detect an effect.

**Table 1:** Characteristics of study sample, overall and cohort-specific

	Total	CZ	FI	FR	IT	NL	PT	ES	SE	UA	UK^a^
Sample size (N)	47,099	2801	6892	1159	637	3387	1843	6486	8308	1757	13 829
Asthma: yes (%)	7.9	1.9	3.2	11.7	8.2	9.1	2.7	4.3	6.7	1.7	14.4
Child’s sex: female (%)	47.9	47.0	49.1	47.0	49.1	48.9	48.6	49.2	48.3	47.0	48.9
Mother’s ethnicity: minority (%)	4.3	0.7	0.0	2.1	2.0	14.5	4.4	2.5	0.0	0.0	8.9
Mother’s age at birth: <30 (%)	50.9	80.4	66.0	46.4	20.7	24.5	35.3	50.4	43.7	84.3	48.6
Firstborn: yes (%)	46.3	48.2	34.3	46.4	58.6	58.0	54.8	57.1	40.2	67.2	43.2
Maternal smoking: yes (%)	21.0	20.4	18.5	19.3	11.0	7.8	29.7	22.5	8.8	10.8	33.0
Mother’s education											
Low (%)	24.1	35.6	25.2	4.2	13.2	8.3	29.7	47.2	33.7	5.2	12.2
Medium (%)	43.5	39.1	51.2	34.9	50.9	20.4	38.9	27.5	32.1	78.4	57.3
High (%)	32.4	25.3	23.7	60.9	36.0	71.3	31.4	25.3	34.2	16.3	30.5

^a^Weighted proportions

**Table 2:** Cohort-specific asthma prevalence, by maternal education level (ISCED levels: low 0-2; medium 3; high 4-6)

	Total asthma		ISCED				
			Asthma %			Test for trend	
	%	(*n*)	Low	Medium	High	*z*	*p*
CZ-CELSPAC	1.93	(54)	2.40	1.46	1.97	0.85	0.396
FI-NFBC	3.18	(219)	3.51	3.15	2.88	1.05	0.294
FR-EDEN	11.65	(135)	10.20	15.59	9.49	2.65	0.008
IT-GASPII	8.16	(52)	8.33	7.41	9.17	−0.53	0.597
NL-ABCD	9.12	(309)	13.83	11.87	7.79	4.35	<0.001
PT-G21	4.33	(281)	4.54	4.88	3.35	1.52	0.128
ES-INMA	6.67	(554)	3.10	2.09	2.94	0.14	0.890
SE-ABIS	2.66	(49)	7.32	6.82	5.88	2.16	0.031
UA-FCOU	1.71	(30)	2.17	1.76	1.40	0.54	0.591
UK-MCS^a^	15.24	(2108)	19.95	15.17	10.59	8.78*	<0.001

^a^Weighted proportions and analysis (unweighted *n*)

*T-value shown. Test for trend similar when replicated using unweighted values

**Fig. 1:**
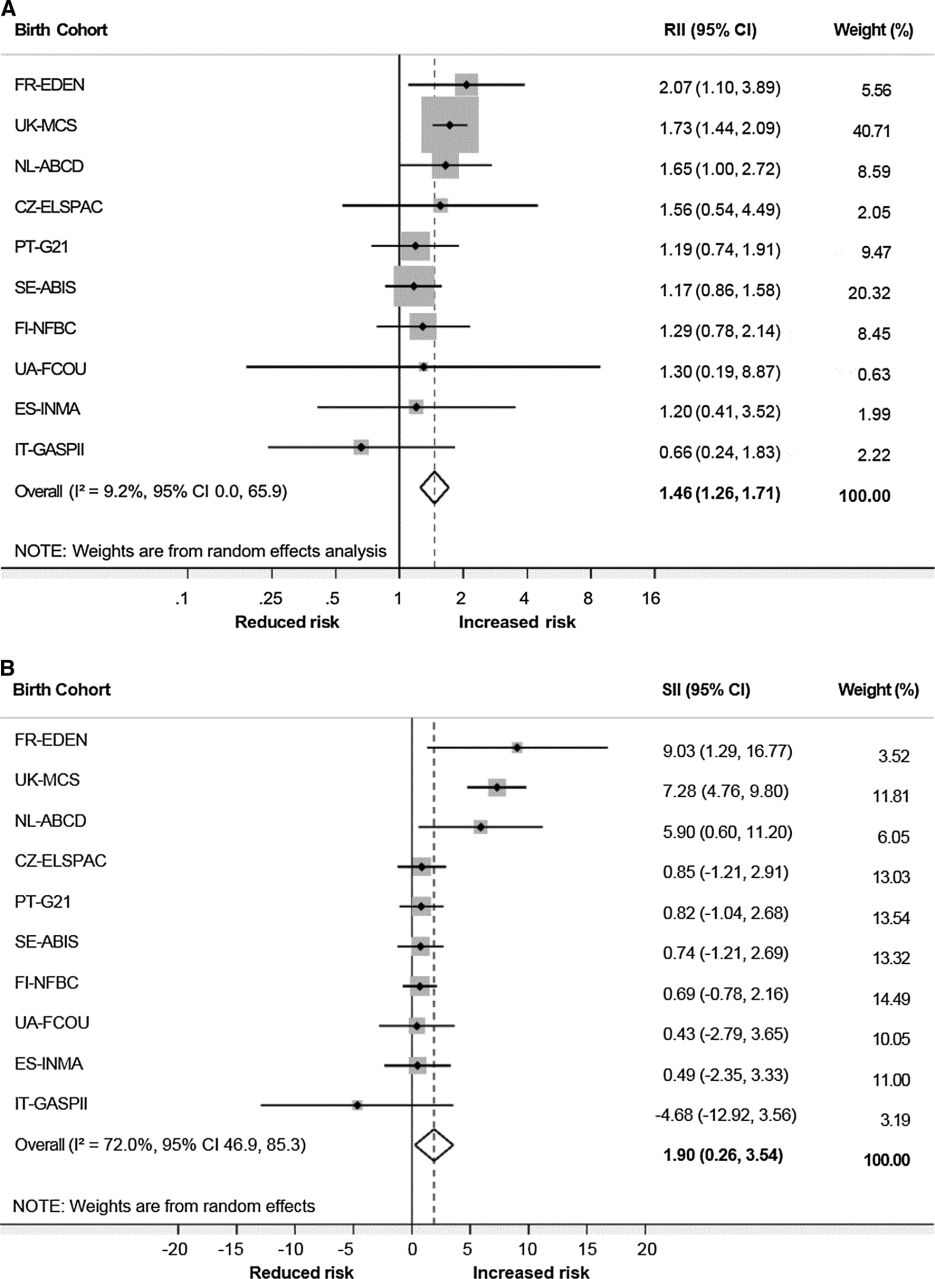
Forest plots of the RII (**a**) and SII (**b**) in childhood asthma across 10 European cohorts (*squares*) and combined (*diamond*), adjusted for child sex, smoking during pregnancy, parity, mother’s age and ethnicity; values to the right of the *undashed vertical line* indicate greater inequity for low compared to high maternal education groups

As displayed in Fig. 1a, the inverse relative risk of asthma with maternal education is strongest in cohorts FR-EDEN (RII 2.07 95% CI 1.10, 3.89), UK-MCS (RII 1.73 95% CI 1.44, 2.09) and NL-ABCD (RII 1.65 95% CI 1.00, 2.72). This pattern is reflected in absolute inequity values (Fig. 1b); Children in the FR-EDEN cohort have a 9.0% risk difference of asthma, UK-MCS a 7.3% difference and NL-ABCD a 5.9% difference. CZ-CELSPAC, FI-NFBC, ES-INMA, PT-G21, SE-ABIS and UA-FCOU have positive RII and SII values, but with smaller statistically insignificant effect sizes. IT-GASPII displays a contrary pattern; low education level is associated with 34% lower odds (RII = 0.66 95% CI 0.24, 1.83) and 4.68% decreased risk difference (SII = -4.68 95% CI -12.92, 3.56) of asthma compared to high education. However, these relationships are not statistically significant.

Across all European cohorts, children born to mothers in the lowest tertile of education were 46% more likely to develop asthma than children born to mothers in the highest tertile (95% CI 1.26, 1.71). The pooled estimate of SII was 1.90 (95% CI 0.26, 3.54), indicating a 1.9% mean risk difference of asthma between children born to mothers in the low compared to high educational group. An *I* ^2^ value of 9.2% (p = 0.36) in the RII model gives evidence of low heterogeneity in relative scores between cohorts; however, absolute scores (SII) were moderately to highly heterogeneous (*I* ^2^ = 72.0%, *p* < 0.001) [28]. Fixed effects meta-analysis techniques slightly increased relative estimates (RII = 1.50 95% CI 1.32, 1.71), whilst decreasing absolute estimates (SII = 1.47, 95% CI 0.70, 2.34).

## Discussion

This study uniquely combined prospective data of educational inequalities in childhood asthma from 10 European cohort studies. Overall, we found relative and absolute asthma risk inversely related to maternal education, which is broadly consistent with previous research [8, 14, 29–32]. However, the magnitude of difference varied across countries and substantial heterogeneity was present in absolute differences. The highest prevalence of asthma and most pronounced inequity were observed in Western European countries.

France, the UK and the Netherlands cohorts had asthma rates between 9 and 14%, compared to less than 2% of the Czech Republic and Ukraine cohorts. East-to-West geographic variations have been noted previously, although are now reportedly diminishing [1]. Whilst climatic risk factors such as low altitude, temperature variations and relative humidity may explain some of the difference, it is argued that the urban ‘western lifestyle’ is a major contributor–and the adoption of this by Eastern Europe the reason for the closing gap [33, 34]. Western lifestyle factors include increased exposure to vehicle emissions and a diet high in calories, fat and sugar, but low in fruit and vegetables.

Maternal education affects child health through several mechanisms. Lower levels of education are linked to lower employment positions, fewer material resources and social support, as well as limited access to health information (and a diminished ability to act on this information) [19, 35, 36]. Prominently, this leads to greater exposure to health damaging risk factors for the mother and consequently their child. Although our analyses were adjusted for several potential risk factors, data constraints meant that many other risk factors were not included. It is likely that a large proportion of inequity may be explained by differences in exposure to risk factors such as tobacco smoke after birth, infant weight gain and adiposity, household damp, infections, and pollution [8–12]. Family history of asthma and atopy, unmeasured in this study, is one of the strongest risk factors for childhood asthma [8]. However, this does not fully explain why the impact of maternal education is greater in some countries compared to others.

Explaining difference in inequity across countries requires us to look at the structural facets that constrain and disadvantage people with lower education [37]. It is proposed that the intergenerational impact of education is additionally conflated by the broader socio-political and cultural landscape within countries [38]. The results from Western Europe suggest that whilst access to health care and social security is important, it is not enough to counter inequities [38]. Universal provision in healthcare, in particular, does not mean universal uptake [29]. For example, disadvantaged groups in the UK are less likely to avail of health promotion and prevention services [39]. Anxo et al.’s [40] review found that the impact of women’s educational attainment on employment integration is more pronounced in France, Italy and Spain compared to Sweden [40]. Higher employment rates and macrosocial policies that value women and families in the Nordic countries may partially explain lower levels of inequity compared to Western Europe [41, 42]. However, high rates of adiposity amongst children of parents with low education persist in Sweden and Finland despite egalitarian policies [37, 43]. The Mediterranean diet may protect against the impact of maternal education in Southern Europe [44].

Birth cohort studies are an essential and unique source of information on the multifaceted predictors of chronic disease [45]. Combining 10 European studies has enabled a comparison of more than 47,000 children over several years of life. The prospective design addresses issues of reverse causation inherent to cross-sectional studies. In addition, as the results were not reliant on published data, this study avoided publication bias. However, several limitations must be acknowledged when considering the current study. Firstly, rates of asthma and the inequities shown are not necessarily indicative of the country or area in which each cohort study were based. Many cohorts sampled only one major urban area and we may expect regional differences in rates of education in rural areas, for instance.

Combining multiple datasets brings difficulties and, as highlighted by the heterogeneous absolute rates of inequity, there is unexplained difference across the included cohorts. Unmeasured confounding, such as the risk factors mentioned above, is likely to contribute to this. In addition, several study aspects were not consistent across cohorts. Age at which asthma was defined ranged from 3 to 8 years old, and the timespan in which to consider a diagnosis from the last 12 months to at any point across the child’s lifetime. Symptomatology of asthma, as well as the impact of inequity on the disease outcome, can differ across age in childhood [1, 14, 46]. Birth year ranged from 1985 to 2008 across the cohorts, adding time-varying contextual factors to the findings. For example, the Spanish cohort study recruited children over a decade, encompassing a period of economic growth and better perinatal outcomes than in the period of economic recession that followed [19].

The proportion of mothers with low education was inconsistent across studies. Both low education and young mothers appear underrepresented in the Italian sample, which may imply limited study power and explain the contradictory results shown by this cohort. Previous cross-sectional research in Rome found a significant trend between lower paternal education and increased risk of asthma, and a similar but insignificant trend of maternal education [47]. The proportion of mothers with low education was also low in France, Ukraine and the Netherlands; however, these are similar to national averages for the time [22]. Highest education level in the Ukraine and Czech Republic cohorts were based on several family members, which likely conflated the education level of some mothers. Almost all cohorts relied on maternal reports of asthma rather than doctor’s diagnosis, which likely led to imprecise estimations of the condition. Cesaroni et al. [47] speculate that parents from higher socio-economic positions are more likely to report asthma especially where symptoms are mild. Missing data for the asthma outcome was significantly related to lower maternal education across the dataset, further indicating a socially patterned bias to the results.

Despite limitations, this comparative analysis across European cohorts demonstrates that maternal education is implicated in offspring asthma risk. The difference in magnitude of inequity across countries, suggests contextual factors affect the translation between maternal education and offspring health outcomes and, importantly, that reduction in inequity is possible. Given that early disadvantage in respiratory health puts into place a negative trajectory that can be difficult to modify [17], these results reinforce the need for early, if not pre-birth, interventions to offset the burden of this disease [18, 38]. Universal and high-quality perinatal care including information about asthma risk that is clearly and respectively articulated is a necessity [48, 49]. Both improving the educational attainment of young women and combatting systematic barriers faced by women with lower education, such as access to employment, is essential to ensure that these offspring are not further disadvantaged [40, 42].

Future cross-European analyses could be improved with the inclusion of known environmental risk factors for asthma and by the use of standardised measures (such as the ISAAC for asthma) to increase comparability and enable pathway analyses [5, 17]. Exclusion of genetic predispositions using genetic methods, such as twin and family studies, will clarify environmental causes with more precision. Differentiating between asthma phenotypes and additional indicators of socioeconomic position, such as income, will help further untangle cross-country differences [29, 50].
